# SIMIT: Subjectively Interesting Motifs in Time Series

**DOI:** 10.3390/e21060566

**Published:** 2019-06-05

**Authors:** Junning Deng, Jefrey Lijffijt, Bo Kang, Tijl De Bie

**Affiliations:** Department of Electronics and Information Systems, Ghent University, Technologiepark-Zwijnaarde 122, 9052 Ghent, Belgium

**Keywords:** time series, motif detection, information theory, subjective interestingness, pattern mining, exploratory data mining

## Abstract

Numerical time series data are pervasive, originating from sources as diverse as wearable devices, medical equipment, to sensors in industrial plants. In many cases, time series contain interesting information in terms of subsequences that recur in approximate form, so-called *motifs*. Major open challenges in this area include how one can formalize the interestingness of such motifs and how the most interesting ones can be found. We introduce a novel approach that tackles these issues. We formalize the notion of such subsequence patterns in an intuitive manner and present an information-theoretic approach for quantifying their interestingness with respect to any prior expectation a user may have about the time series. The resulting interestingness measure is thus a *subjective* measure, enabling a user to find motifs that are truly interesting *to them*. Although finding the best motif appears computationally intractable, we develop relaxations and a branch-and-bound approach implemented in a constraint programming solver. As shown in experiments on synthetic data and two real-world datasets, this enables us to mine interesting patterns in small or mid-sized time series.

## 1. Introduction

There exists a myriad of data mining methods for time series data, ranging from fully-automated change detection, classification, and prediction methods, to exploratory techniques such as clustering and motif detection. Change and motif detection are related in the sense that local patterns (motifs) and longer-running changes in the profile of the time series need to be evaluated against a prior that specifies what the expected profile is, typically in the form of a probability distribution.

Prior work on time series motif detection tended to evaluate a motif’s interestingness by assessing its significance against some objectively-chosen prior distribution for the time series (either explicitly or implicitly). The result is that the most “interesting” motifs found are often trivial, implied by the user’s prior expectations. In contrast to this, we introduce an approach to identify recurring subsequence patterns that are *subjectively interesting*, i.e., interesting when contrasted with the user’s prior expectations. A recurring subsequence is a subsequence that is found at several positions within the time series with some variation, and this will be called a *motif*.

To achieve this, we define subsequence patterns as local probabilistic models. The subjective interestingness of a subsequence pattern is then defined in terms of the amount of information (in an information-theoretic sense) contained in this local model, when contrasted with a *background distribution* that represents the user’s expectations. Initially, the background distribution is computed as the distribution of maximum entropy subject to any prior user expectations as constraints, such as constraints on the expected mean, variance, and co-variance between neighboring points in the time series. Upon revealing the presence of a subsequence pattern, the background distribution is updated to account for this new knowledge, such that it continues to represent the (now updated) expectations of the user as subsequence patterns are revealed throughout an iterative analysis. The amount of information gained by the time series can be computed by contrasting the prior distribution and the updated distribution.

To find the most informative motifs and outliers efficiently, we develop relaxations and propose an effective search algorithm implemented in a constraint programming solver. Together with an additional heuristic pruning technique, this enables one to mine subsequence patterns relatively efficiently.

Our specific contributions are:

- Novel definitions of motifs as probabilistic patterns (Section 3).
- A quantification of their Subjective Interestingness (SI), based on how much information a user gains when observing this pattern (Section 4).
- A relaxation of the exact setting and an algorithm to efficiently mine the most interesting subsequence patterns for a user (Section 5).
- Several speedup techniques that result in a computationally more efficient algorithm (Section 6).
- Empirical evaluation of this algorithm on one synthetic dataset and two real-world datasets, to investigate its ability to encode the user’s prior beliefs and identify interesting subsequence patterns (Section 7).

## 2. Related Work

Time series motifs usually hint at useful information about seasonal or temporal associations between events, and detecting such patterns can be very useful in practice. A myriad of techniques for motif discovery have been proposed. These can be categorized from different perspectives, starting with the definition of the interestingness measure for a motif. In general, two main aspects for judging the interestingness of a motif exist in the literature, namely the similarity among instances and the support (i.e., the number of instances in a motif) [1]. More specifically, one prioritizes a motif whose instances exhibit maximum similarity, or even more strictly, defining a motif as the most similar subsequence pair (e.g., [2,3,4]); whereas the second prioritizes one with the highest support given a minimum similarity between all instances of a motif (e.g., [5,6]).

For existing work adopting either similarity-based or support-based interestingness, the similarity measure plays a key role in the motif discovery algorithms, and typical ones include Euclidean distance and dynamic time warping. Regarding the massive computational cost, some efforts have been made to represent time series in low dimensional space. Examples of such representations include Symbolic Aggregate Approximation (SAX), DFT, and random projections. A review of motif discovery algorithms based on their similarity measure and representation was provided by Mueen [1].

In addition to these aspects, there exist several challenging issues in this pattern discovery problem, including scalability [3,7,8], the detection of motifs with various lengths [9,10], multi-dimensional time series [11], coping with streaming data [12,13], and handling distortions [14]. For a more comprehensive review of existing publications regarding these issues, we refer the interested readers to Torkamani and Lohweg [15].

Our work explores a new aspect, shedding light on the essence of the interestingness for a motif, which we believe depends on a user’s prior knowledge. Previous measures that prioritized either the similarity or support were all objective. However, for a user with prior information about the time series (a common situation), the resulting motifs may be trivial. Hence, we propose a novel subjective interestingness measure, which enables ones to identify motifs that contradict their prior expectations and are truly interesting to them. Additionally, the information-theoretic view that we take immediately provides a balance between the similarity and numerosity for a set of subsequences to form a motif.

## 3. Motifs and Motif Templates

We denote a *time series* as $\hat{x}≜{\left({\hat{x}}_{1},\ldots ,{\hat{x}}_{n}\right)}^{\prime }\in R^{n}$, i.e., an ordered collection of *n* real numbers ${\hat{x}}_{i}\in R$, where i∈[n]=[1,…,n]. We write ${\hat{x}}_{i,l}$ for $\hat{x}$ for the *subsequence* of length l≤n−i+1 starting from position *i*. That is, ${\hat{x}}_{i,l}≜{\left({\hat{x}}_{i},\ldots ,{\hat{x}}_{i+l-1}\right)}^{\prime }\in R^{l}$. By sliding a window of size *l* along $\hat{x}$ and extracting each subsequence, we can obtain a set containing all the subsequences of length *l*. We denote this set as $S_{l}$, i.e., $S_{l}=\left\{{\hat{x}}_{i,l}|i=1,2,\ldots ,n-l+1\right\}$. Note that hatted symbols represent empirical values, and their non-hatted equivalents are used to denote the respective random variables.

### 3.1. Motif

A *motif* of length *l* denoted by $T_{l}$ is a subset of $S_{l}$ containing more than two non-overlapping subsequences. That is, $T_{l}\subseteq S_{l}$, $|T_{l}|\geq 2$, |i−j|≥l,$\forall {\hat{x}}_{i,l},{\hat{x}}_{j,l}\in T$, and i≠j.

Each subsequence in a motif is said to be an instance of the motif. As we focus on identifying motifs of a fixed length (i.e., *l*), we write T for $T_{l}$ in the rest of the paper for convenience. Not every motif is equally interesting. The criterion by which we judge the quality of a motif is explained below.

The index set of a motif T is denoted as $I_{T}$, i.e., $I_{T}=\left\{i|{\hat{x}}_{i,l}\in T\right\}$.

### 3.2. Motif Template

Our general aim is to find subjectively interesting “motifs”. However, what one typically means is not actually a set of subsequences that are similar, but a general subsequence pattern that is recurring in a time series. To avoid working with a set of subsequences, one could use a single exemplar. Here, we introduce a probabilistic local model as the target object, the *motif template*, instead.

**Definition** **1** (Motif template)**.** *A motif template is a probability distribution over the space of motif instances, i.e., $R^{l}$.*

More concretely, we propose a template where we capture the mean and variance statistics of instances and call this a *mean-variance motif template*. We deem the roles played by these two statistics essential, as the mean serves as a figure about the motif shape, and the variance tells the extent of the similarity among these instances. A typical choice of model is a multivariate Gaussian distribution parameterized by the mean and variance statistics.

It is in principle straightforward to also use covariance statistics, but such a model has $O(l^{2})$ parameters and is not interpretable. Thus, we define a *mean-variance motif template* as:

**Definition** **2** (Mean-variance motif template)**.** *A mean-variance motif template is a multivariate Gaussian distribution N(μ,Σ) over the space of motif instances. Σ is the diagonal matrix with the values of standard deviations as the main diagonal and zero elsewhere. Hence, this distribution can be essentially parameterized by a tuple (μ,σ), where μ is a vector of means and σ is a vector of standard deviations, both of length l.*

**Figure 1:**
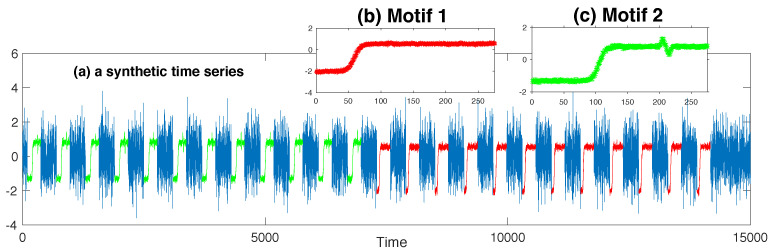
The algorithm correctly retrieves the two patterns in the synthetic data.

**Figure 2:**
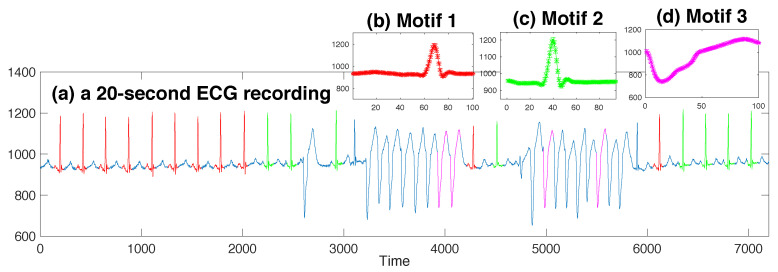
Three motif templates identified in the 20-second ECG recording.

**Figure 3:**
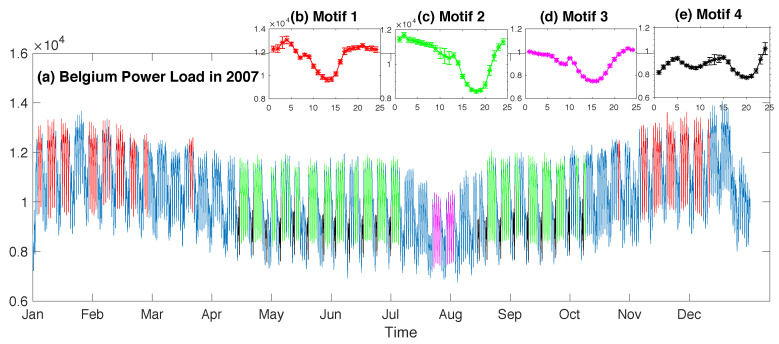
Four motif templates identified in the Belgium power load data.

In this paper, we take μ,σ as the maximum likelihood parameters over the set of instances in a motif. We denote the parameter tuple for the motif template learned from the motif T as $\left(\mu_{T},\sigma_{T}\right)$. That is, $\mu_{T}=\frac{1}{\left|T\right|}\sum_{i\in I_{T}}{\hat{x}}_{i,l}$, $\sigma_{T}=\frac{1}{|T|-1}\sum_{i\in I_{T}}{\left({\hat{x}}_{i,l}-\mu_{T}\right)}^{2}$. Examples are given in Figure 1, Figure 2 and Figure 3.

## 4. Formalizing the Subjective Interestingness

Previous motif discovery work tended to quantify the interestingness in an objective way (see Section 2). For a data analyst with prior knowledge about the time series, which we believe is common, the discovered patterns may be trivial to the end user and could be easily implied. To preempt this, we propose to use a more flexible subjective measure of interestingness.

### 4.1. The Background Distribution

We follow the so-called FORSIED (an acronym for “Formalizing Subjective Interestingness in Exploratory Data mining”) framework [16,17] to quantify the subjective interestingness of a motif. The basic procedure is that a *background distribution* is defined over the space of all possible datasets, which here would be all possible realizations of a time series x. Since $x\in R^{n}$, the background distribution is defined by a probability density function *p*. The background distribution essentially encodes the beliefs and expectations of the user about the data. More specifically, it assigns a probability density to each possible data value according to how tenable the user thinks this value is. It was argued that a good choice for the background distribution is the maximum entropy distribution subject to constraints that capture the user’s prior expectations about the data.

#### 4.1.1. The Initial Background Distribution

We wish to define constraints and compute a maximal entropy distribution such that these constraints are preserved in expectation. For the initial background distribution, we consider three kinds of constraints. They respectively express the user’s prior knowledge about the mean and the variance of each data point, as well as the first order difference in x. Notice that these expectation values can be anything; here, we equate them to the empirical values. With these three constraints, the initial background distribution is the solution to Problem 1 as stated as follows:

**Problem** **1.** (1) $$\begin{array}{cc} \max_{p} & \int -p(x)\log(p(x))dx, \end{array}$$

(2) $$\begin{array}{cc} s.t. & \int p\left(x\right)\frac{1}{n}\sum_{i=1}^{n}x_{i}dx=\hat{m}1, \end{array}$$

(3) $$\begin{array}{c} \int p\left(x\right)\frac{1}{n}\sum_{i=1}^{n}{\left(x_{i}-\hat{m}\right)}^{2}dx=\hat{v}1, \end{array}$$

(4) $$\begin{array}{c} \int p\left(x\right)\frac{1}{n-1}\sum_{i=1}^{n-1}{\left(x_{i}-x_{i+1}\right)}^{2}dx=\hat{d}1, \end{array}$$

$$\mathrm{where}:\;\hat{m}=\frac{1}{n}\sum_{i=1}^{n}{\hat{x}}_{i},\;\hat{v}=\frac{1}{n}\sum_{i=1}^{n}{\left({\hat{x}}_{i}-\hat{m}\right)}^{2},\hat{d}=\frac{1}{n-1}\sum_{i=1}^{n-1}{\left({\hat{x}}_{i}-{\hat{x}}_{i+1}\right)}^{2}.$$

and 1 is an *n*-dimensional vector with all the entries as one.

The solution to Problem 1 is a multivariate Gaussian distribution parameterized by an *n*-dimensional mean vector m and an n×n covariance matrix V. The values of m and V can be derived by applying the Lagrange multiplier method. We further improve the computation efficiency by using the property that maximizing the entropy and maximizing the likelihood are the dual of each other in the class of exponential form distributions [18]. The computation details are given in Appendix A.

#### 4.1.2. Updating the Background Distribution

Once a motif template along with its instances is identified and shown to the user, the user’s belief state changes, and the background distribution needs to be updated. The background distributions *p* for all prior belief types discussed in this paper are essentially multivariate Gaussian distributions each of which is parametrized by m and V. As mentioned, the motif template is also described by a multivariate Gaussian distribution, $N(\mu_{T},\Sigma_{T})$. To make the updated background distribution reflect the user’s newly-acquired knowledge, we simply set the blocks of current m and V corresponding to the subsequence instances equal to $\mu_{T}$ and $\Sigma_{T}$ and the off-diagonal elements of V corresponding to instances equal to zero. We denote the background distribution having incorporated T as $p_{T}$.

### 4.2. A Remark about No Independence Assumption

**Remark** **1.** *We do not assume independence between time points. While in the local motif model (i.e., the mean-variance motif template), time points are indeed independently distributed (see Section 3.2), this is not the case for the model of the whole time series x (indeed, the full covariance matrix is not necessarily diagonal). Moreover, it is important to realize that the background distribution is a model for the user’s belief state; it is not a model for the stochastic source of the data. In other words, if the background distribution does not exhibit a certain dependency, this does not mean that the data may not come from a stochastic source that exhibits this dependency. It only means that the user whose belief state is modeled by this background distribution is not yet aware of it. As the covariance matrix is not diagonal, it is indeed the case that updating the expected value even for a single point in the time series can ripple across the sequences and modify the expected values throughout.*

### 4.3. The Subjective Interestingness Measure

Intuitively, a good motif is one whose instances are strongly similar to each other and together account for a considerable portion on the whole time series. Consider such a good motif T. If all instances are similar to each other, it directly follows that the values of $\mu_{T}$ are similar to those of each instance, and the diagonal entries of $\Sigma_{T}$ are small. After revealing the motif to the user, the background distribution is updated to be $p_{T}$. Since the parameters of $p_{T}$ consist of $\mu_{T}$ and $\Sigma_{T}$, the new background distribution $p_{T}$ will thus be a more accurate model for the time series. More precisely, the probability of the data under $p_{T}$ is larger. To quantify the amount of information *gained* by the motif, we can compare this probability to the one under the previous background distribution *p*. The more strongly they differ, the more this motif enhances with the user’s beliefs about the data.

Mathematically, we define the Information Content (IC) of a motif as the difference between the log probability for the whole time series $\hat{x}$ under $p_{T}$ and that under *p*:

(5) $$\mathrm{IC}\left(T\right)=\log p_{T}\left(\hat{x}\right)-\log p\left(\hat{x}\right).$$

The rationale is that minus the log probability of the data represents the number of bits of information the data contain with respect to the probability distribution, so this difference corresponds to the amount of information (in bits) the user has gained by seeing the motif.

Note that the expected value of IC(T) with respect to $p_{T}\left(\hat{x}\right)$ takes the same form as the Kullback–Leibler divergence, but this does not mean that IC and KL-divergence are equivalent concepts. The KL-divergence measures the difference between two probability distributions, but here, the $p_{T}\left(\hat{x}\right)$ and $p(\hat{x})$ in the definition of IC(T) are probabilities rather than distributions.

### 4.4. Finding the Most Subjectively Interesting Motif Template

Now, we can formalize our goal of finding the most interesting motif in a time series as an optimization problem with the following objective:

$$\mathrm{Objective}\;1:\;\;\underset{T}{\mathrm{argmax}}\log p_{T}\left(\hat{x}\right)-\log p\left(\hat{x}\right).$$

Objective 1 accounts for the probability of the whole of the data. This probability depends on the parameter updating of *p* (i.e., m and V) from incorporating subsequences and can thus embody the quality of the choice for template instances. Note that the key changes of m and V only take place on the part of their entries that represent instances in T. That means, the rise in the probability of the whole of the data is mostly related to the probability of those instances in T. Based on this observation, we propose a relaxed version of Objective 1, which only depends on the probability of instances in T. This objective is similar to Objective 1, but is more straightforward to optimize efficiently.

$$\mathrm{Objective}\;2:\;\;\underset{T}{\mathrm{argmax}}\sum_{i\in I_{T}}\log p_{T}\left({\hat{x}}_{i,l}\right)-\sum_{i\in I_{T}}\log p\left({\hat{x}}_{i,l}\right).$$

## 5. Method

In this work, we adopted a greedy search algorithm to identify the most interesting motif. The general idea is to first seed T by finding a small set of *k* instances according to Objective 2 and then greedily grow that set using Objective 1.

The algorithm consists of three major steps:

1. Model the user’s prior belief by the initial background distribution;
2. Seed by finding a small set of instances that optimizes Objective 2;
3. Grow that set by adding an instance that optimizes Objective 1 and iterate.

**Remark** **2.** *Although the three basic steps are for finding a single motif (i.e., the most interesting one to the user), our algorithm is not limited to that. A new search for another motif can be triggered by running Step 2 and Step 3 again based on an updated background distribution, the one that has already incorporated the user’s knowledge of the previous motif.*

How we compute the initial background distribution (i.e., Step 1) is described in the above (see Section 4.1.1). In the following, we go into more details of Steps 2 and 3.

### 5.1. Step 2: Finding a Seed Motif $T^{\left(0\right)}$ with k Instances

The search starts by finding *k* non-overlapping optimal instances that constitute a seed set for T. We denote such a seed set by $T^{\left(0\right)}$. The most subjectively interesting $T^{\left(0\right)}$ is identified by optimizing Objective 2. This problem can be formulated as:

**Problem** **2.** (6) $$\begin{array}{c} \underset{T^{\left(0\right)}}{\mathrm{argmax}}\sum_{i\in I_{T^{\left(0\right)}}}\log p_{T^{\left(0\right)}}\left({\hat{x}}_{i,l}\right)-\sum_{i\in I_{T^{\left(0\right)}}}\log p\left({\hat{x}}_{i,l}\right)\equiv \\ \underset{T^{\left(0\right)}}{\mathrm{argmax}}\sum_{i\in I_{T^{\left(0\right)}}}\log N\left({\hat{x}}_{i,l}|\mu_{T^{\left(0\right)}},\Sigma_{T^{\left(0\right)}}\right)-\sum_{i\in I_{T^{\left(0\right)}}}\log N\left({\hat{x}}_{i,l}|m^{\left(i:i+l-1\right)},V^{\left(i:i+l-1,i:i+l-1\right)}\right),\\ \mathrm{where}\;\mu_{T^{\left(0\right)}}=\frac{1}{k}\sum_{i\in I_{T^{\left(0\right)}}}{\hat{x}}_{i,l},\;\Sigma_{T^{\left(0\right)}}=\mathrm{diag}\left(\frac{1}{k-1}\sum_{i\in I_{T^{\left(0\right)}}}{\left({\hat{x}}_{i,l}-\mu_{T^{\left(0\right)}}\right)}^{2}\right). \end{array}$$

The superscript of a vector or matrix symbol is used to denote the corresponding entry. Using the expression for the multivariate Gaussian distribution, we can write Equation (6) as:

(7) $$\begin{array}{cc} & \sum_{i\in I_{T^{\left(0\right)}}}\log N\left({\hat{x}}_{i,l}|\mu_{T^{\left(0\right)}},\Sigma_{T^{\left(0\right)}}\right)-\sum_{i\in I_{T^{\left(0\right)}}}\log N\left({\hat{x}}_{i,l}|m^{\left(i:i+l-1\right)},V^{\left(i:i+l-1,i:i+l-1\right)}\right)\\ = & -\frac{kl}{2}\log\left(2\pi \right)+\frac{kl}{2}\left\{\log k+\log(k-1)\right\}\\ & \underset{I}{\underset{︸}{-\frac{k}{2}\sum_{h\in [l]}\log\left\{\sum_{\begin{array}{c} i,j\in I_{T^{\left(0\right)}}\\ i<j \end{array}}{\left({\hat{x}}_{i,l}^{\left(h\right)}-{\hat{x}}_{j,l}^{\left(h\right)}\right)}^{2}\right\}}}-\frac{1}{2}\left(k-1\right)l\\ & \underset{II}{\underset{︸}{-\sum_{i\in I_{T^{\left(0\right)}}}\log N\left({\hat{x}}_{i,l}|m^{\left(i:i+l-1\right)},V^{\left(i:i+l-1,i:i+l-1\right)}\right)}}. \end{array}$$

Note that the parts related to the choice of instances in $T^{\left(0\right)}$ are underbraced and numbered respectively as I and II. By taking a closer look, we can see that part II is essentially the sum of all the individual negative log probabilities of ${\hat{x}}_{i,l}$ under *p*, and the values for parameters m and V are not subject to which instances to incorporate. This allows gaining some computational benefits by simply pre-computing each log probability. Nevertheless, I expresses a mutual relationship among all the instances in $T^{\left(0\right)}$, due to its being in the summation form for the logarithm of a summation. Pre-computation is not trivial, which makes the search for optimal instances computationally demanding, reaching $O(n^{k}k^{2})$. We thus adopted a strategy to mitigate a certain factor of this time complexity, as well as a heuristic to prune the search space. A detailed description is provided in Section 6.

### 5.2. Step 3: Greedily Searching for a New Instance

The algorithm then continues to search for a new subsequence that optimizes Objective 1. The search stops when no new subsequence exists such that incorporating it can increase the probability of the time series under the background distribution, i.e., ∄i∈[n−l+1] s.t. $T\cup \{x_{i,l}\}$ is a motif and $p_{T\cup \{x_{i,l}\}}\left(\hat{x}\right)-p_{T}\left(\hat{x}\right)\geq 0$.

To gain some speedup, we pruned subsequences that posed little potential according to a heuristic (see Section 6).

## 6. Speedup Techniques

In this section, we describe some speedup techniques applied to Step 2 (Section 6.1) and Step 3 (Section 6.2).

### 6.1. Speeding Up Step 2

#### 6.1.1. Strategy 1: Bounding Objective 2 and Finding the Submatrix with the Maximal Sum

Recall only terms I and II in the objective of Problem 2 (i.e., Equation (7)) are affected by the chosen instances for $T^{\left(0\right)}$, and the term I makes the search computationally expensive. To mitigate the time complexity, we considered optimizing a relaxed objective of Problem 2 based on bounding the term I. Via applying Jensen’s inequality [19], the term I can be upper bounded by a summation form taken from all the instance pairs:

(8) $$\begin{array}{cc} I: & -\frac{k}{2}\sum_{h\in [l]}\log\left\{\sum_{\begin{array}{c} i,j\in I_{T^{\left(0\right)}}\\ i<j \end{array}}{\left({\hat{x}}_{i,l}^{\left(h\right)}-{\hat{x}}_{j,l}^{\left(h\right)}\right)}^{2}\right\}\\ \leq & \underset{III}{\underset{︸}{\sum_{\begin{array}{c} i,j\in I_{T^{\left(0\right)}}\\ i<j \end{array}}\left\{-\frac{1}{k-1}\sum_{h\in [l]}\log\left\{{\left({\hat{x}}_{i,l}^{\left(h\right)}-{\hat{x}}_{j,l}^{\left(h\right)}\right)}^{2}\right\}\right\}}}-\frac{kl}{2}\log\left\{\frac{k(k-1)}{2}\right\}. \end{array}$$

Substituting Equation (8) into Equation (7) yields:

(9) $$\begin{array}{cc} & \sum_{i\in I_{T^{\left(0\right)}}}\log N\left({\hat{x}}_{i,l}|\mu_{T^{\left(0\right)}},\Sigma_{T^{\left(0\right)}}\right)-\sum_{i\in I_{T^{\left(0\right)}}}\log N\left({\hat{x}}_{i,l}|m^{\left(i:i+l-1\right)},V^{\left(i:i+l-1,i:i+l-1\right)}\right)\\ \leq & -\frac{kl}{2}\log\left(2\pi \right)+\frac{kl}{2}\left\{\log k+\log(k-1)\right\}\\ & +\underset{III}{\underset{︸}{\sum_{\begin{array}{c} i,j\in I_{T^{\left(0\right)}}\\ i<j \end{array}}\left\{-\frac{1}{k-1}\sum_{h\in [l]}\log\left\{{\left({\hat{x}}_{i,l}^{\left(h\right)}-{\hat{x}}_{j,l}^{\left(h\right)}\right)}^{2}\right\}\right\}}}-\frac{kl}{2}\log\left\{\frac{k(k-1)}{2}\right\}\\ & -\frac{1}{2}\left(k-1\right)l\underset{II}{\underset{︸}{-\sum_{i\in I_{T^{\left(0\right)}}}\log N\left({\hat{x}}_{i,l}|m^{\left(i:i+l-1\right)},V^{\left(i:i+l-1,i:i+l-1\right)}\right)}}. \end{array}$$

Finding the maximal value for the objective (Equation (9)) is essentially the same as maximizing term III+ term II. Then, we constructed a matrix $\hat{M}$, with rows and columns representing subsequence candidates, the *i*th diagonal entry ${\hat{M}}_{i,i}$ being the part of the term II inside the summation (Equation (11) below) and the entry at the *i*th row and *j*th column ${\hat{M}}_{i,j}$ being the part of the term III inside the outer summation (Equation (12) below).

Solving Problem 2 corresponds to finding the upper triangular matrix inside $\hat{M}$ with the maximum sum, as expressed in the following problem:

**Problem** **3.** (10) $$\begin{array}{c} \underset{T^{\left(0\right)}}{\mathrm{argmax}}\sum_{i\in I_{T^{\left(0\right)}}}\sum_{j\in I_{T^{\left(0\right)}}}{\hat{M}}_{i,j}, \end{array}$$

(11) $$\begin{array}{c} \mathrm{where}\;{\hat{M}}_{i,i}=\log N\left({\hat{x}}_{i,l}|m^{\left(i:i+l-1\right)},V^{\left(i:i+l-1,i:i+l-1\right)}\right)\;\mathrm{for}\;i\in I_{T^{\left(0\right)}}, \end{array}$$

(12) $$\begin{array}{c} \;\;\;{\hat{M}}_{i,j}=-\frac{1}{k-1}\sum_{h\in [l]}\log\left\{{\left({\hat{x}}_{i,l}^{\left(h\right)}-{\hat{x}}_{j,l}^{\left(h\right)}\right)}^{2}\right\}\;\mathrm{for}\;i,j\in I_{T^{\left(0\right)}}, \end{array}$$

(13) $$\begin{array}{c} \;\;\;\;T^{\left(0\right)}\subseteq \mathrm{PrunedSubsequenceSet}, \end{array}$$

(14) $$\begin{array}{c} \;\;\;|i-j|\geq l,\;\;\forall i,j\in I_{T^{\left(0\right)}}\;\mathrm{and}\;i\neq j. \end{array}$$

The fourth set of constraints (Equation (14)) is to ensure instances in $T^{\left(0\right)}$ are non-overlapping with each other. This speedup technique enables us to compute the matrix $\hat{M}$ in advance and then do the search using Constraint Programming (CP). The time complexity of a relaxed Problem 2 is $O(n^{k})$, a factor of $k^{2}$ less than Problem 2. Clearly, it still appears intractable for real-world applications. To counter this, we deliberately reduced the search space so that each element of $T^{\left(0\right)}$ was constrained to be in a pruned range, denoted by PrunedSubsequenceSet (Equation (13)). The way we constructed PrunedSubsequenceSet is described in the following.

#### 6.1.2. Strategy 2: Pruning

The exhaustive search for a solution to the relaxed Problem 2 is still computationally demanding for a large $\hat{M}$. We thus adopted a heuristic strategy so that the search was in a considerably reduced space, but the quality of the found motifs was guaranteed.

It appears that an off-diagonal entry at the *i*th row and *j*th column ${\hat{M}}_{i,j}$ models a sort of similarity between the subsequence ${\hat{x}}_{i,l}$ and ${\hat{x}}_{j,l}$. As the transition property of the similarity suggests, if ${\hat{M}}_{i,v}$ and ${\hat{M}}_{j,v}$ are large, then so is ${\hat{M}}_{i,j}$. We can deduce that all the entries in $\hat{M}$ mapped from $I_{T^{\left(0\right)}}$ should have a relatively larger value. Hence, we can deliberately perform the search in a pruned range of subsequences whose indices corresponding to largest entries in $\hat{M}$. Specifically, we fixed the first instance to be a certain subsequence and searched the others among subsequences corresponding to the largest 1% entries at a row of $\hat{M}$ corresponding to this instance (i.e., pruning factor =99%). To find the globally optimal *k* instances for $T^{\left(0\right)}$, we fixed the first instance to be each possible subsequence and solved the relaxed *Problem* 2 each time. The final solution should be the one that leads to the maximal objective value.

### 6.2. Speeding Up Step 3

In Step 3, the exhaustive search for a new optimal instance requires checking the result of Objective 1’s value for incorporating every possible subsequence, which is apparently time consuming for large $\hat{x}$. Clearly, incorporating subsequences that bear strong similarity with instances in $T^{\left(0\right)}$ can result in a high value for Objective 1. As the off-diagonal entries in $\hat{M}$ encode a similarity between subsequence pairs, we applied a heuristic pruning strategy based on entries in $\hat{M}$ to reduce the search space.

Assume we are in the stage of having incorporated all the *k* instances in $T^{\left(0\right)}$. Let us denote the current $\hat{M}$ as ${\hat{M}}^{k}$. The new optimal instance must be among those that can produce a relatively large value of the objective for Problem 3, but based on ${\hat{M}}^{k+1}$, whose entry at the *i*th row and *j*th column (i≠j) is $-\frac{1}{k}\sum_{h\in [l]}\log\left\{{\left({\hat{x}}_{i,l}^{\left(h\right)}-{\hat{x}}_{j,l}^{\left(h\right)}\right)}^{2}\right\}=\frac{k-1}{k}{\hat{M}}_{i,j}^{k}$ (recall that ${\hat{M}}_{i,j}^{k}$ is computed by Equation (12)). The objective for Problem 3 is in the form of summing some entries of ${\hat{M}}^{k+1}$ that correspond to instances in $T^{\left(0\right)}$ and the new subsequence (e.g., ${\hat{x}}_{r,l}$). Thus, the potential of ${\hat{x}}_{r,l}$ (i.e., $\mathrm{Potential}({\hat{x}}_{r,l})$) can be captured by how much the value of the objective for Problem 3 (Equation (10)) increases if incorporating ${\hat{x}}_{r,l}$:

$$\mathrm{Potential}\left({\hat{x}}_{r,l}\right)=\sum_{i\in I_{T^{\left(0\right)}}}{\hat{M}}_{r,i}^{k+1}+{\hat{M}}_{r,r}^{k+1}.$$

The algorithm ranks all possible subsequences in descending order according to their potential values. Then, the search is implemented in a greatly reduced space (i.e., among those in the top 1% of the rank). Let us denote the optimal subsequence that leads to the highest Objective 1 value by $T_{k+1}$. We first check whether the probability of the time series increases under the new background distribution. If so, we include $T_{k+1}$ in T. Nevertheless, for incorporating the next subsequence, a further check is performed. First, we update the search domain, as well as the potential rank by deleting all the subsequences overlapped with $T_{k+1}$. We do Step 3 to identify the new optimal one. If this subsequence is still among the top three of the potential rank and incorporating it did not trigger the stop condition, we make it the (k+2)th instance of T. Otherwise, we recompute the potential and rank all the subsequences again, according to ${\hat{M}}_{k+2}$, the one considering $T_{k+1}$ as an incorporated instance. Then, Step 3 is done again among an updated search domain. However, there might occur a situation where the new optimal one is still not ranked among the top three. In this case, if the stop condition is not reached, we make it an instance of T anyway. By this lazy greedy strategy, the search space is significantly reduced, while a good quality of the incorporated instance is ensured to a satisfiable extent.

## 7. Experiments

This section describes the evaluation of our proposed algorithm on a synthetic and two real-world datasets. In the following, we first describe the datasets (Section 7.1). Second, we empirically analyze how the pruning percentage in the initial set selection affects the quality of the result in terms of the SI (Section 7.2). Then, we discuss the properties of the discovered motifs in each dataset to assess their validity (Section 7.3).

All experiments were conducted on a PC with Ubuntu OS, Intel(R) Core(TM) i7-7700K 4.20-GHz CPUs, and 32 GB of RAM. The main algorithm was implemented in MATLAB R2016b. The step of identifying the initial motif template was coded in Python 3.5, in which the open source software *OR-Tools* 6.10 [20] developed by Google was used as the constraint programming solver. All the computer codes are available at https://bitbucket.org/ghentdatascience/simit-public/src.

### 7.1. Data

- **Synthetic time series:** We synthesized a time series of length 15,000. This series included 2 sorts of motif trends, and their prototypes were taken from 2 subsequence instances in the UCRTrace Data [21]. Both instances were of the same length as 275, but belonged to different classes. Subsequences for each motif were generated by sampling from a Gaussian distribution with the mean as the corresponding instance and a reasonably small variance as 0.01. There were in total 12 subsequences for each motif. The remaining were standard Gaussian noises, and they constituted a major part of the whole series. More details about the data synthesizing process are described in the pseudocode Procedure 1 in Appendix B.
- **MIT-BIHarrhythmia ECG recording:** This dataset was Recording #205 in the MIT-BIH Arrhythmia DataBase [22]. This recoding was created from digitizing the ECG signals at 360 samples per second. We chose a part of 20 s (7200 samples) to experiment on that included normal heartbeats and ventricular tachycardia beats.
- **Belgium Power Load Data:** This dataset was taken from *Open Power System Data* [23]. The primary source of these data was ENTSO-E Data Portal/Power Statistics [24]. *Open Power System Data* then resampled and merged the original data in a large CSV file with hourly resolution. The part we selected to experiment on recorded the total load in Belgium during the year 2007, for a total length of 24×365=8760.

### 7.2. Pruning and Scalability

For all the experiments, we first identified an initial motif $T^{\left(0\right)}$ with k=4 instances. As mentioned above, our algorithm searches among a space pruned in a particular heuristic way to gain some relative amount of efficiency. The effects of pruning in the initial set identification were tested on the synthetic time series, for which the correct answers were known. The results indicated that the optimal one was still found even with the heaviest pruning (99.9%). Therefore, we used 99% pruning in the experiments on the real-world datasets. The scalability of our algorithm, with respect to the length of the motif template and to the length of the time series, was evaluated on the ECG recording. Table 1 shows that the length of the motif template did not influence the computational cost that much, but the influence of the time series length was more than quadratic.

### 7.3. Results

#### 7.3.1. Synthetic Data

In this experiment, we specified the length of the motif instance the same as the length of the subsequence synthesized by sampling (i.e., l=275). As expected, our algorithm identified two motifs embedded in this synthetic time series, the result of which is shown in Figure 1. The whole time series is plotted in Figure 1a, and subsequences incorporated into the first motif set are exactly those sampled from the same Gaussian distribution (in red). Figure 1b illustrates the first motif template by plotting the mean of all the instances incorporated into this motif, as well as the error bars indicating the variance of each point. Our algorithm also correctly identified the second motif (marked in green in Figure 1a). We modeled the user’s knowledge about the first motif by triggering the new search on a new original background distribution, the one that took into account of all the instances for the first motif. The second motif is displayed in Figure 1c.

#### 7.3.2. ECG Time-Series

We analyzed the ECG data by identifying motifs with length 100, corresponding to a duration of 0.28 s. In this fairly short recording (see Figure 2a), our algorithm identified three motifs. The first two motifs corresponded to normal heartbeats (highlighted with red and green; templates shown in Figure 2b,c). We can see that their shapes mostly coincide, with a horizontal shift. Normal heart beats are deemed to be similar to each other, but within each one, there may exist a particular subsection that bears more similarity than other subsections. Since the motif length was set to be less than a period of a normal heart beat, our algorithm was prone to regarding those subsections that bore the similarity to a different extent to be in different motif sets. Another motif identified by the algorithm lied in the area of the ventricular tachycardia (pink sections). The instances did not cover all the ventricular tachycardia heart beats, but the small error bars in Figure 2d indicate that these instances were uncannily similar to each other, and the reason why other ventricular tachycardia subsequences lost the membership for this motif set was their smaller similarity.

#### 7.3.3. Belgium Power Load Data

We analyzed these data searching for motifs of length 24 (one day). The first four motifs discovered by our algorithm are displayed in Figure 3. The first motif covered many weekdays, except for Fridays, during cold seasons (highlighted with red in Figure 3a). All these 24-h periods started at 15:00. Note that not all the Mondays–Thursdays during these months were identified as the motif instance, for example those blue sections both at the very beginning and the end of this whole series. The reason could be that they corresponded to holidays rather than normal workdays. As for other workdays in winter excluding Friday that did not belong to this motif, these are very interesting for energy analysts to analyze their reason. After modeling the user’s knowledge about this motif, our algorithm then identified the second motif, corresponding to Monday–Thursday as well, but during hot seasons (highlighted with green in Figure 3a). Most days in July were not instances of this motif. This might be due to them being during summer holiday time (a noticeable blue and pink section that divides the green section in Figure 3a). Actually, part of these days (i.e., Monday–Thursday in the last two weeks of July) constituted the third motif (pink sections in Figure 3a). The first 3 motifs were all related to normal workdays excluding Friday, but in different temperature conditions. It seems that power consumption in hot seasons is less regular than that during cold seasons, as the normal workday pattern relating to cold periods was identified first (i.e., the first motif). This phenomenon could be very interesting for energy analysts to investigate. By incorporating these 3 motifs into the user’s belief model, our algorithm identified the fourth motif, corresponding to some Sundays from the middle of April to the beginning of October (black sections in Figure 3a). All the instances belonging to the same motif corresponded to daytime starting at exactly the same hour, and they were strongly similar to each other, as reflected by the small error bars in the illustration of each motif template (Figure 3b–d).

## 8. Conclusions

We proposed a new methodology for motif discovery called SIMIT (for Subjectively Interesting Motifs in Time Series) and a concrete implementation for a specific type of motifs where the interestingness score can incorporate prior beliefs, and hence are subjectively interesting. We developed a relaxation of this interestingness score with bounds that can be optimized relatively efficiently using constraint programming. An empirical evaluation demonstrates the potential of the proposed approach.

For future work, it would be useful to develop a motif template that incorporates a form of time warping. Secondly, the length of the subsequences considered is currently a parameter and could be optimized as well. To make this possible, further speedup techniques should be developed. In contrast to motifs, *outliers* are subsequences that are unusual and nonrecurring in a time series. Identifying subjective outliers can also be interesting. Moreover, the proposed motif templates are based on multivariate Gaussian distributions. An extension to multivariate non-Gaussian distributions [25] with the use of non-symmetrical entropy [26] seems promising. Finally, an extension towards multivariate time series is useful.

## Figures and Tables

**Table 1:** Run-time to search the initial motif set, with a pruning factor of 99%.

n	l	Time (s)		n	l	Time (s)		n	l	Time (s)
1800	100	9.96		3600	100	50.12		7200	100	369.92
7200	25	328.09		7200	50	350.65		7200	100	369.92

## Appendix A. Solving Problem 1

The solution of Problem 1 follows directly from applying the method of *Lagrange multipliers*. Let us use Lagrange multipliers $\lambda_{1},\lambda_{2},\lambda_{3}$ for constraints (Equations (2)–(4)), respectively, λ for the vector containing all Lagrange multipliers. The Lagrangian is formulated as:

(A1) $$\begin{array}{cc} L\left(\lambda ,p(x)\right)= & -\int p(x)\log p(x)dx\\ & +\lambda_{1}\left(\int p\left(x\right)\frac{1}{n}\sum_{i=1}^{n}x_{i}dx-\hat{m}1\right)+\lambda_{2}\left(\int p\left(x\right)\frac{1}{n}\sum_{i=1}^{n}{\left(x_{i}-\hat{m}\right)}^{2}dx-\hat{v}1\right)\\ & +\lambda_{3}\left(\int p\left(x\right)\frac{1}{n-1}\sum_{i=1}^{n-1}{\left(x_{i}-x_{i+1}\right)}^{2}dx-\hat{d}1\right). \end{array}$$

Differentiating Equation (A1) w.r.t. p(x) and renormalizing by dropping the dx factor yields:

(A2) $$\begin{array}{cc} \frac{\partial }{\partial p(x)dx}L\left(\lambda ,p(x)\right)= & -\log p(x)-1\\ & +\lambda_{1}\left(\frac{1}{n}\sum_{i=1}^{n}x_{i}\right)+\lambda_{2}\left(\frac{1}{n}\sum_{i=1}^{n}{\left(x_{i}-\hat{m}\right)}^{2}\right)\\ & +\lambda_{3}\left(\frac{1}{n-1}\sum_{i=1}^{n-1}{\left(x_{i}-x_{i+1}\right)}^{2}\right). \end{array}$$

Equating Equation (A2) to zero and solving for p(x) gives:

(A3) $$p\left(x\right)=\frac{1}{Z}\exp\left\{\lambda_{1}\left(\frac{1}{n}\sum_{i=1}^{n}x_{i}\right)+\lambda_{2}\left(\frac{1}{n}\sum_{i=1}^{n}{\left(x_{i}-\hat{m}\right)}^{2}\right)+\lambda_{3}\left(\frac{1}{n-1}\sum_{i=1}^{n-1}{\left(x_{i}-x_{i+1}\right)}^{2}\right)\right\}.$$

where *Z* is the normalization variable, as the implicit normalization constraint ∫p(x)dx=1 can be imposed constructively by setting *Z* to be:

$$Z=\int \exp\left\{\lambda_{1}\left(\frac{1}{n}\sum_{i=1}^{n}x_{i}\right)+\lambda_{2}\left(\frac{1}{n}\sum_{i=1}^{n}{\left(x_{i}-\hat{m}\right)}^{2}\right)+\lambda_{3}\left(\frac{1}{n-1}\sum_{i=1}^{n-1}{\left(x_{i}-x_{i+1}\right)}^{2}\right)\right\}dx.$$

By expanding quadratic terms in Equation (A3) and reorganizing, we obtain:

$$\begin{array}{cc} p\left(x\right)=\frac{1}{Z}\exp\{ & \left(\lambda_{2}+\lambda_{3}\right)x_{1}^{2}+\sum_{i=2}^{n-1}\left(\lambda_{2}+2\lambda_{3}\right)x_{i}^{2}+\left(\lambda_{2}+\lambda_{3}\right)x_{n}^{2}\\ & +\sum_{i=1}n\left(\lambda_{1}-2\lambda_{2}\hat{m}\right)x_{i}-\sum_{i=1}^{n-1}2\lambda_{3}x_{i}x_{i+1}+\lambda_{2}n{\hat{m}}^{2}\}. \end{array}$$

After some algebra:

(A4) $$p\left(x\right)=\frac{1}{Z}\exp\left\{\frac{1}{2}{\left(x-m\right)}^{T}V^{-1}\left(x-m\right)\right\}.$$

$$\begin{array}{cc} \mathrm{where}\;m & =\hat{m}1\\ V_{1,1}^{-1} & =V_{n,n}^{-1}=\lambda_{2}+\lambda_{3},V_{i,i}^{-1}=\lambda_{2}+2\lambda_{3}\;\mathrm{for}\;i=2,\ldots ,n-1\\ V_{i,j}^{-1} & =-\lambda_{3}\;\mathrm{for}\;|i-j|=1,V_{i,j}^{-1}=0\;\mathrm{for}\;\left|i-j\right|>2,\\ \lambda_{1} & =0. \end{array}$$

As is clearly seen from Equation (A4), p(x) is essentially a multivariate Gaussian distribution with the mean vector m and the covariance matrix V. Note that the inverse of the covariance matrix $V^{-1}$, known as the *precision matrix*, in our case is a tridiagonal matrix whose nonzero elements can be determined by $\lambda_{2}$ and $\lambda_{3}$. As maximizing the entropy and maximizing the likelihood are the dual of each other in the class of exponential families [18], the optimal values of the Lagrange multipliers $\lambda_{2},\lambda_{3}$ can be found by maximizing the likelihood over the observations $L(\hat{x}|\lambda )$. This problem can be formulated as:

**Problem** **A1.**

$$\lambda^{*}=\underset{\lambda }{\mathrm{argmax}\;}L\left(\hat{x}|\lambda \right)$$

Problem A1 is convex and can be solved by using standard techniques for convex optimization (e.g., the interior point method [27]).

## Appendix B. Pseudocode for Generating the Synthetic Data

**Procedure 1:** Synthetic time series generation.
**input**: Trace Instance 1, Trace Instance 2 **output**: A synthesized time series $\hat{x}$ **1** n←15000 // The length of the synthesized time series; **2** l←275 // The length of each subsequence in a motif whose prototype is taken from Trance Instance 1 or 2; **3** S← An n×n diagonal matrix with each diagonal entry as 0.001; **4** $Q_{\mathrm{prototype}1}\leftarrow$ The set containing the beginning indices for 12 subsequences for Prototype 1; **5** $Q_{\mathrm{prototype}2}\leftarrow$ The set containing the beginning indices for 12 subsequences for Prototype 2; **6** $Q_{\mathrm{others}}\leftarrow$ The set containing indices that are not covered by subsequences for Prototype 1 or 2; **7** // Generating subsequences for Prototype 1 by sampling( **8** **for** $i\in Q_{\mathrm{prototype}1}$ **do** **9** ⌊(${\hat{x}}_{i,l}∼N\left(\mathrm{Trace}\;\mathrm{Instance}\;1,S\right)$; **10** // ( Generating subsequences for Prototype 2 by sampling( **11** **for** $i\in Q_{\mathrm{prototype}2}$ **do** **12** ⌊(${\hat{x}}_{i,l}∼N\left(\mathrm{Trace}\;\mathrm{Instance}\;2,S\right)$; **13** // ( Making the remaining standard Gaussian noises( **14** **for** $i\in Q_{\mathrm{others}}$ **do** **15** ⌊(${\hat{x}}_{i,1}∼N\left(0,1\right)$

## References

[1] Mueen A. (2014). Time series motif discovery: dimensions and applications. Wiley Interdiscip. Rev. Data Min. Knowl. Discov..

[2] Mueen A., Keogh E.J., Zhu Q., Cash S., Westover M.B. (2009). Exact Discovery of Time Series Motifs.

[3] Yeh C.M., Zhu Y., Ulanova L., Begum N., Ding Y., Dau H.A., Silva D.F., Mueen A., Keogh E. Matrix Profile I: All Pairs Similarity Joins for Time Series: A Unifying View That Includes Motifs, Discords and Shapelets. Proceedings of the 2016 IEEE 16th International Conference on Data Mining (ICDM).

[4] Mueen A., Chavoshi N. (2015). Enumeration of time series motifs of all lengths. Knowl. Inf. Syst..

[5] Lin J., Keogh E., Lonardi S., Patel P. Finding Motifs in Time Series. Proceedings of the ACM SIGKDD.

[6] Chiu B., Keogh E., Lonardi S. Probabilistic Discovery of Time Series Motifs. Proceedings of the ACM SIGKDD.

[7] Rakthanmanon T., Campana B.J.L., Mueen A., Batista G.E.A.P.A., Westover M.B., Zhu Q., Zakaria J., Keogh E. Searching and mining trillions of time series subsequences under dynamic time warping. Proceedings of the ACM SIGKDD.

[8] Yoon C.E., O’Reilly O., Bergen K.J., Beroza G.C. (2015). Earthquake detection through computationally efficient similarity search. Sci. Adv..

[9] Senin P., Lin J., Wang X., Oates T., Gandh S., Boedihardjo A.P., Chen C., Frankenstein S. (2018). GrammarViz 3.0: Interactive Discovery of Variable-Length Time Series Patterns. ACM TKDD.

[10] Linardi M., Zhu Y., Palpanas T., Keogh E. Matrix Profile X: VALMOD—Scalable Discovery of Variable-Length Motifs in Data Series. Proceedings of the SIGMOD.

[11] Yeh C.M., Kavantzas N., Keogh E. Matrix Profile VI: Meaningful Multidimensional Motif Discovery. Proceedings of the IEEE ICDM.

[12] Mueen A., Keogh E. Online Discovery and Maintenance of Time Series Motifs. Proceedings of the ACM SIGKDD.

[13] Lin J., Li Y. Finding approximate frequent patterns in streaming medical data. Proceedings of the IEEE International Symposium on CBMS.

[14] Keogh E., Wei L., Xi X., Lee S., Vlachos M. LB_Keogh Supports Exact Indexing of Shapes under Rotation Invariance with Arbitrary Representations and Distance Measures. Proceedings of the 32nd International Conference on Very Large Data Bases.

[15] Torkamani S., Lohweg V. (2017). Survey on time series motif discovery. Wiley Interdiscip. Rev. Data Min. Knowl. Discov..

[16] De Bie T. An information-theoretic framework for data mining. Proceedings of the ACM SIGKDD.

[17] De Bie T. Subjective interestingness in exploratory data mining. Proceedings of the IDA.

[18] De Bie T. (2011). Maximum entropy models and subjective interestingness: an application to tiles in binary databases. Data Min. Knowl. Discov..

[19] Jensen J.L.W.V. (1906). Sur les fonctions convexes et les inégalités entre les valeurs moyennes. Acta Math..

[20] Google Google Optimization Tools(OR-Tools). https://github.com/google/or-tools.

[21] Chen Y., Keogh E., Hu B., Begum N., Bagnall A., Mueen A., Batista G. (2015). The UCR Time Series Classification Archive. www.cs.ucr.edu/eamonn/timeseriesdata/.

[22] Moody G.B., Mark R.G. (2001). The Impact of the MIT-BIH Arrhythmia Database. IEEE Eng. Med. Biol. Mag..

[23] Open Power System Data (2018). Data Package Time Series. https://data.open-power-system-data.org/time_series/.

[24] ENTOSO-E Detailed Hourly Load Data for All Countries 2006–2015. https://www.entsoe.eu/data/data-portal/.

[25] Contreras-Reyes J.E. (2015). Renyi entropy and complexity measure for skew-Gaussian distributions and related families. Phys. A Stat. Mech. Appl..

[26] Liu C. (2009). Nonsymmetric entropy and maximum nonsymmetric entropy principle. Chaos Solitons Fractals.

[27] Potra F.A., Wright S.J. (2000). Interior-point methods. J. Comput. Appl. Math..

